# Enhancing Medical Spanish Education and Proficiency to Bridge Healthcare Disparities: A Comprehensive Assessment and Call to Action

**DOI:** 10.7759/cureus.48512

**Published:** 2023-11-08

**Authors:** Alexandra Lopez Vera

**Affiliations:** 1 Medical Education, California University of Science and Medicine, Colton, USA

**Keywords:** health policy, racial concordance, language concordance, medical education, medical spanish

## Abstract

This article highlights the critical importance of linguistic and cultural concordance in health care, particularly in addressing the shortage of proficient Spanish-speaking healthcare providers in California. It advocates for standardized curricula, qualified instructors, and mandatory medical Spanish courses while stressing the significance of interdisciplinary training that integrates language skills with clinical experience and acknowledges the interplay between language and culture in health care. The article calls for proactive efforts from medical schools, faculty, and healthcare providers, emphasizing standardized curricula, culturally sensitive training, and reliable assessment tools. Additionally, it underscores the need to enhance the representation of underrepresented minority healthcare providers to ensure equitable health care for linguistic minorities, emphasizing the shared responsibility of healthcare and education stakeholders.

## Editorial

The need for healthcare providers that are linguistically and culturally concordant is essential to ensure equitable and high-quality health care for patients with non-English language preferences. In medical consultations, a significant level of linguistic competence is often indispensable. Without effective communication, tasks such as collecting a patient's medical history, establishing a diagnosis, and creating a well-monitored treatment plan become challenging [1]. Research has indicated that patients experience higher levels of satisfaction when they receive care from healthcare providers who speak the same language, highlighting the significant impact of linguistic concordance on healthcare quality and patient well-being [2]. There are two main approaches to addressing language-concordant care for patients with limited English proficiency (LEP). The first one has to do with assigning a professional medical interpreter to the patient, and the second one with matching a patient with a language-concordant healthcare provider [3].

To effectively deliver language-appropriate services to linguistic minorities, healthcare providers or medical interpreters should take into account the language behaviors of the specific population they are serving [4]. In the United States, approximately 25 million people have non-English language preferences, with the majority being Spanish speakers. Nearly 30% of these individuals reside in California [5]. Even for patients who prefer languages other than English and possess some proficiency in English, conveying health-related information in English can still present communication challenges [4].

There is still a shortage of primary care physicians fluent in Spanish [6]. Due to the widespread use of volunteer and makeshift interpreters in clinical settings lacking professional interpreters, patients with LEP may face communication barriers when seeking medical care, which can lead to misunderstandings, misdiagnoses, suboptimal treatment [7], and a higher risk of readmission [8]. Thus, prioritizing language concordance is a crucial step in promoting health equity for the expanding Spanish-speaking patient population, the well-being and effectiveness of the physicians attending to them, and the overall quality of healthcare within the US health system [9].

This study presents a series of recommendations aimed at addressing the pressing issue of a shortage of proficient Spanish-speaking healthcare providers in California. While focusing on California, a state with a significant number of Spanish-speaking residents, the strategies outlined in this article are extendable to other states and regions with dense Spanish-speaking populations. By delving into both the advancements achieved thus far and the obstacles faced in this particular context, the study seeks to shed light on the multifaceted challenges of bridging language and cultural gaps in medical settings. The aim is to reduce healthcare disparities by providing future healthcare professionals with the vital skills to effectively navigate the complex cultural and linguistic challenges that can arise when working with Spanish-speaking patients, as well as with interpreters when their services are needed.

The current state of medical Spanish education in California medical schools

Section 1557 of the Affordable Care Act (ACA) has had a significant impact on regulations related to medical language services. This legislation mandates that healthcare providers and health insurance companies receiving federal funding are required to provide qualified interpreters to patients with LEP [10]. In 2015, California faced a significant shortage of interpreters, with only 738 certified medical interpreters available to assist 1.7 million individuals with LEP, creating a challenge for health centers to meet the demand for interpreter services [11]. Furthermore, only a minority of patients with LEP in culturally and linguistically diverse regions receive healthcare interpreters. These patients may express dissatisfaction with their healthcare experiences, often preferring direct communication with healthcare providers and over-relying on interpreters [2].

Medical schools, responsible for nurturing proficient healthcare practitioners, face the challenge of preparing students to navigate complex communication from medical schools through residency and independent practice [9].

To address the shortage of Spanish-speaking healthcare providers, many medical schools in California offer medical Spanish education aimed at improving patient-physician communication for the growing Spanish-speaking demographic. However, when medical Spanish courses lack essential standards concerning curriculum organization, qualified instructors, student evaluation, and institutional recognition, they may boost students' self-confidence without significantly enhancing their linguistic skills, potentially exacerbating communication issues with patients from language-minority backgrounds [12].

Several medical schools in California offer specialized medical Spanish courses to equip their students with essential language skills for healthcare contexts. Stanford University School of Medicine, Loma Linda University, and Touro College of Osteopathic Medicine provide medical Spanish elective courses through online platforms. Meanwhile, the University of California at San Francisco, Los Angeles, San Diego, and Riverside offer practical courses designed to cultivate foundational skills for overcoming cultural and linguistic barriers in health care. Additionally, Kaiser Permanente School of Medicine, Western University College of Pharmacy, and California Health Sciences University College of Osteopathic Medicine provide Essential Medical Spanish courses, all available as elective options. These programs aim to enhance students' capacity to communicate directly with Spanish-speaking patients, contributing to improved patient care.

The California University of Science and Medicine (CUSM) stands out as a pioneer among California's medical schools. Beginning in the academic year 2024-2025, CUSM will introduce mandatory medical Spanish courses for its Medical Degree (MD) students. Situated 60 miles east of Los Angeles, CUSM's vision centers on establishing a socially accountable medical school that prioritizes education, research, and service, focusing on the healthcare needs of its community and fostering students to excel as empathetic physicians, scientists, and leaders [13]. This initiative marks a significant step in preparing future healthcare providers with the necessary cultural and linguistic proficiency to serve the Spanish-speaking patient population effectively. The Medical Spanish program (Vida) combines didactic and hands-on elements in medical Spanish education, tailored to the needs of Southern California's Hispanic populations. It goes beyond traditional classroom instruction, offering clinical experience to address the need for effective assessment methods in medical language courses [14]. In addition, it also integrates physician-patient communication skills, fostering a synergistic learning experience through weekly medical Spanish sessions and interactions with trained Spanish-speaking standardized patients (SPs). By emphasizing cultural sensitivity and addressing implicit bias through comprehensive in-person sessions, the Vida program equips students with the essential cross-cultural communication skills to bridge the gap between language and culture in health care [14].

Navigating obstacles in California's medical Spanish education

Other medical schools attempting to replicate the success of CUSM's mandatory medical Spanish courses may face considerable challenges. These obstacles may arise due to resistance from both medical faculty and students who might not immediately perceive the benefits of introducing required medical Spanish courses and may argue that such additional language requirements impose an additional burden on already busy medical students. The effective integration of medical Spanish courses into pre-clinical medical school curricula, particularly in institutions located in densely populated Spanish-speaking regions, should be seen as a valuable goal [15]. To successfully implement these required medical Spanish courses, institutions must commit to providing qualified faculty and substantial resources for curriculum development, language practice, and clinical experiences. Developing a curriculum that not only teaches the language but also effectively integrates language skills with clinical experiences and patient interactions is a complex and resource-intensive endeavor, but it is essential for addressing the critical issue of linguistic and cultural concordance in health care and ensuring equitable access to quality healthcare services for Spanish-speaking communities [14].

Even if medical Spanish courses were to be nationally required in medical schools, it is essential to recognize that this measure alone may prove insufficient for adequately preparing healthcare providers to deliver medical care effectively to Spanish-speaking communities. This assertion is supported by a substantial body of evidence that consistently reveals healthcare disparities associated with factors such as race and ethnicity across a wide spectrum of medical conditions and healthcare services [16]. Hence, improving the representation of underrepresented minority healthcare providers in the long term should be a key objective, as it not only improves access but also elevates the quality of care provided [17]. In the short term, addressing the linguistic dimension in isolation may fail to target the fundamental sources of these disparities. Thus, it is imperative to underscore the inseparable relationship between language and culture. Healthcare providers must not only possess proficiency in the language but also have a deep understanding of the cultural context within which health care is delivered to genuinely bridge the gap in healthcare disparities for Spanish-speaking communities.

Assessment strategies for strengthening medical Spanish education

A limited number of medical Spanish programs adhere to best practices, primarily because of the absence of a reliable method for assessing both individual student performance and overall program effectiveness [18]. Many medical schools in the state incorporate Objective Structured Clinical Examinations (OSCEs) in Spanish, along with practice sessions involving Spanish monolingual and Spanish-English bilingual SPs. OSCEs offer a distinctive opportunity to evaluate student language proficiency within a high-fidelity clinical context, replicating clinical scenarios in which learners interact with trained SPs. Currently, no validated tool exists for assessing student language proficiency in non-English OSCEs [19].

In the realm of assessment, a notable challenge revolves around the perceived readiness of medical Spanish educators when it comes to evaluating students' proficiency in this specialized language. Within US medical schools, educators specializing in medical Spanish come from diverse backgrounds in terms of training and qualifications. The majority of these educators are either physicians (78%) or language professors [19]. This interdisciplinary nature of the field of medical Spanish underscores the pressing need to provide training to educators in areas of medical Spanish to which they may not have previously been formally exposed [12-19].

Medical Spanish assessment tools encompass student self-assessment, oral proficiency interviews, or the Interagency Language Roundtable (ILR) scale, modified for healthcare purposes [20]. The ILR scale has proven to be as accurate as an oral proficiency interview, but its validity often extends to individuals who self-assess at the lower or higher ends of the scale [21]. The need for a comprehensive assessment tool that empowers professionals to independently engage with Spanish-speaking populations becomes apparent when considering the lack of options for determining intermediate proficiency levels [3].

Undoubtedly, the presence of a medical interpreter plays a vital role in facilitating effective communication within healthcare settings. Extensive research indicates that engaging professional interpreters, individuals who have completed rigorous training and certification, is linked to significant enhancements in the overall quality of care provided to patients with LEP [22]. An analysis of 48 hospitals across the United States revealed several of these facilities were lacking crucial resources, such as Spanish-translated patient materials, medical interpreters, or Spanish-speaking healthcare providers [23]. Moreover, it highlighted the importance of utilizing bilingual clinicians when available [23]. Therefore, one potential strategy could be to involve training healthcare providers and medical students to become proficient in the Spanish language. Nevertheless, it is essential to acknowledge that healthcare providers, while theoretically capable of serving as interpreters for their colleagues, primarily bear the responsibility of patient care, diagnosis, and treatment. As such, relying on them for interpreting purposes is suboptimal, given their primary healthcare duties. Additionally, assessing Spanish proficiency for healthcare providers in a medical context transcends standard written or oral examinations of basic Spanish skills. It necessitates the incorporation of evaluations that encompass patient interaction, cultural awareness, and medical terminology knowledge, all of which are integral to medical practice [9]. Hence, it is not advisable to train healthcare providers as medical interpreters, as this could divert their focus from their primary responsibilities.

To meet the pressing demand for a dependable assessment instrument for evaluating medical professionals' oral proficiency in Spanish, a collaborative team of language experts and medical practitioners has adeptly adapted the Student Oral Language Observation Matrix (SOLOM). This tool has been modified for its application in clinical contexts, employing video-recorded OSCE interactions involving medical students and Spanish-speaking SPs. This refined and innovative assessment tool is now known as the Physician Oral Language Observation Matrix (POLOM) [3].

To ascertain its reliability, a panel of raters extensively assessed medical professionals' performance using the POLOM [3]. The study used 356 video-recorded SP encounters from a medical Spanish course at the University of Illinois College of Medicine spanning from 2013 to 2020. These encounters involved standardized scenarios such as pelvic pain, upper abdominal pain, chest pain, and shortness of breath. A bilingual rating team consisting of physicians and linguists with advanced Spanish proficiency assessed the encounters. Results demonstrated that experienced raters can offer highly reliable scores, a crucial milestone in the programmatic development of the POLOM as a tool for assessing clinical Spanish language proficiency [3]. The author's goal is to validate the POLOM in future studies, ultimately becoming the first standardized rating tool for medical oral language proficiency in a non-English language.

Assessing medical students with detailed, category-specific oral proficiency evaluations carries considerable significance within the realm of medical Spanish education. Such assessment mechanisms, which pinpoint individual strengths and weaknesses, facilitate the customization of educational programs to address specific challenges and cater to the unique needs of each student. Furthermore, the reliability demonstrated by this new assessment instrument, even when administered by a single well-trained rater, underscores its adaptability for use by either an experienced physician or a linguist rater [3].

Call to action

It is imperative for medical schools to proactively address the shortage of proficient Spanish-speaking healthcare providers. To effectively tackle this issue, these institutions should consider implementing mandatory medical Spanish courses that not only boost students' confidence in the language but also genuinely enhance their linguistic skills. This enhancement should be complemented by maintaining consistent curriculum organization, ensuring the presence of qualified instructors, and establishing rigorous evaluation processes. Furthermore, these courses should transcend traditional classroom instruction by providing practical, hands-on clinical experiences that bridge the gap between language acquisition and real-world patient interactions. These experiences are instrumental in better preparing students to communicate effectively with Spanish-speaking patients, comprehend their needs, and deliver high-quality healthcare services.

In addition to language proficiency, it is essential to recognize the inseparable relationship between language and culture in health care. It should be a priority for medical faculty to provide future healthcare providers with not only language proficiency but also a deep understanding of the cultural context within which health care is delivered. Cross-cultural training should be integrated into medical education to bridge the gap in healthcare disparities for Spanish-speaking communities. Robust evaluation processes must also be instituted to assess students' language proficiency and cultural competency, thereby ensuring their preparedness to serve Spanish-speaking communities and contribute to the reduction of health disparities in these populations.

Supporting the development and adoption of reliable assessment tools for evaluating students' language proficiency in Spanish OSCEs is another vital step. Medical schools should be encouraged to use tools like the POLOM to assess clinical Spanish language proficiency, thereby ensuring that healthcare providers are well-prepared to serve Spanish-speaking patients effectively.

Finally, while improving language skills is essential, it should be complemented by efforts to enhance the representation of underrepresented minority healthcare providers. This not only improves access but also elevates the quality of care provided to diverse patient populations. The imperative to provide equitable health care to linguistic minorities is a responsibility shared by medical schools, medical faculty, language faculty, and healthcare providers.
